# Thiazolidinediones and Fertility in Polycystic Ovary Syndrome (PCOS)

**DOI:** 10.1155/PPAR/2006/73986

**Published:** 2006-12-14

**Authors:** Pascal Froment, Philippe Touraine

**Affiliations:** ^1^INSERM Unité 418, UMR Communications Cellulaire et Différenciation, Hôpital Debrousse, 29 Rue Soeur Bouvier, 69322 Lyon, France; ^2^Department of Endocrinology and Reproductive Medicine, GH Pitié-Salpêtrière, 47-83 Boulevard de l’Hôpital, 75651 Paris Cedex 13, France

## Abstract

Polycystic ovary syndrome (PCOS) is the most frequent cause of female infertility. The treatment of PCOS patients with insulin sensitizers, such as metformin or thiazolidinediones, increases the ovulation rate and the number of successful pregnancies. The positive action of the insulin-sensitizing treatments could be explained by a decrease in the peripheral insulin resistance but also by a direct action at the ovarian level. We report in this review different hypotheses of thiazolidinediones actions to improve PCOS (steroid secretion by ovarian cells ; insulin sensitivity in muscle and adipocyte and fat redistribution).

## INTRODUCTION

Polycystic ovary syndrome (PCOS) is the most frequent cause of female infertility, affecting about 5–10% of women in age of procreation [1]. Diagnostic criteria to establish PCOS are controversial [1, 2], involving two among the following three 2003 Rotterdam's criteria: first, clinical and/or signs of hyperandrogenism, second a chronic absence of ovulation and finally, third, the increase of ovarian volume and/or the presence of at least 12 follicles in the 2- to 9-mm range in each ovary, detected by ultrasonography [3]. Moreover, insulin resistance is a common metabolic feature associated with PCOS, up to 50–70% of patients in some series [4].

Yen et al described a vicious circle where several endocrine abnormalities could maintain the PCOS status [2, 5]. Three entrance points are proposed as follows.

1. Alteration of the hypothalamo-pituitary axis (∼ 50% cases) [6] with high circulating LH levels that can lead to excess androgens and contribute to the formation of cystic follicles, as described in the mouse model [7]. However, the importance of this hypothesis as an entrance point is critical in the PCOS syndrome [8].
2. Hyperandrogenism due to steroidogenic dysregulation in thecal cells. Mutations of Cyp11a1 or Cyp17 genes are detected in some patients and could lead to an hyperactivity of the steroidogenesis [9, 10].
3. As evidenced by Franks et al, hypersensitivity of ovarian cells to both insulin and gonadotropin leads to androgens hypersecretion [11].

**Figure 1:**
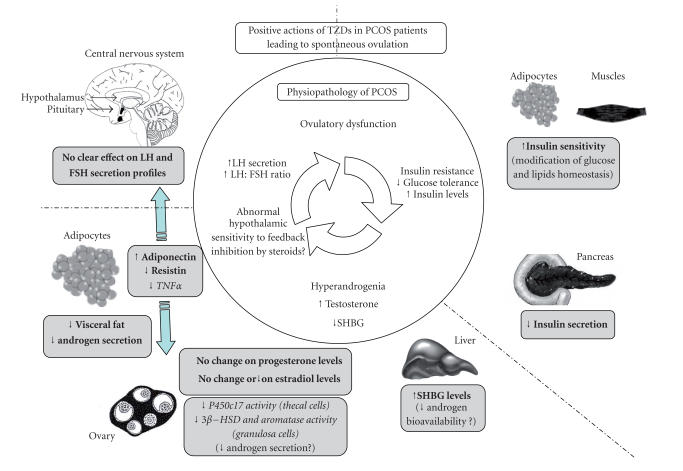
Positive actions of TZDs in PCOS patients leading to spontaneous ovulation. Three entrance points/major endocrine abnormalities (hyperandrogenia, insulin resistance, or LH hypersecretion) lead or maintain the ovulatory dysfunction of PCOS as represented in the inner circle of the figure. For example, high LH concentrations or insulin increase androgen secretion by human thecal cells [12] and could contribute to impair follicular development. In addition, elevated androgens could reduce hypothalamic sensitivity to negative steroids feedback, because administration of flutamide, an antiandrogen, can restore this sensitivity [13]. The positive actions of TZDs on PCOS patients could be mainly at two levels as described in the outer part of the figure. (1) TZDs increase the insulin sensitivity and decrease the insulin secretion. (2) TZDs reduce the androgen secretion and/or activity (in ovary and/or in adipose tissue). Finally, TZDs can modulate secretion of several endocrine hormones (adiponectin, resistin, TNF*α*) which can reduce androgen production or improve gonadotropin secretion. The boxes show results obtained in vivo in PCOS women (in bold) or in vitro in human cell culture (in italics).

The treatment of PCOS patients with insulin sensitizers of various drug families, such as thiazolidinediones (TZDs), metformin or D-chiro-inositol, increases the ovulation rate and the number of successful pregnancies (cf [14–17]). The positive action of these “insulin sensitizers” drugs could be explained by various manners. We report in this review different hypothesis of TZDs actions to improve PCOS at each level of the “Yen vicious circle” (Figure 1).

TZDs are synthetic ligands also known as glitazones (troglitazone, rosiglitazone or pioglitazone) [18], which can bind and activate the nuclear receptor, peroxysome proliferator-activated receptor gamma (PPAR*γ*) (cf [19, 20]). PPAR*γ* could be considered as a fuel sensor linking the energy metabolism and reproduction to inform cells on the energy status. Indeed, PPAR*γ* can regulate the transcription and/or activity of different key regulators of energy homeostasis [19] such as glucose or lipid regulators (PPAR*γ* upregulated expression of glucose transporters, insulin receptor, insulin receptor substrate, fatty acid-binding protein, etc) (cf [21]). Activation of PPAR*γ* by TZDs increases insulin sensitivity mainly in adipocytes and muscle cells [22], and also stimulates the differentiation of adipose cells (cf [23, 24]).

In addition, the three PPAR isoforms (PPAR*α*, PPAR*β*/*δ*, PPAR*γ*) are expressed along the gonadotrope axis (central nervous system, pituitary gland and ovary) (cf [25, 26]). In the ovaries, expression of PPAR*γ* is restricted to follicles, primarily to granulosa cells in developing follicles, slightly in theca cells and in corpus luteum (cf [25]). After the LH surge, the PPAR*γ* expression decreases in follicle [27, 28]. In general, it is considered that TZDs activate PPAR*γ*, nevertheless a PPAR*γ*-independent action of TZDs cannot be excluded, as suggested by several recent studies [29, 30].

## ASSESSMENT OF THE CLINICAL TRIALS OF TZDS TREATMENTS

Pioglitazone and rosiglitazone are the unique TZDs which could be used. Indeed, troglitazone was withdrawn from the worldwide market in 2000 because of its hepatotoxicity. Pioglitazone and rosiglitazone possess mainly the same properties, except that pioglitazone may have a more positive effect on lipid profile than rosiglitazone [31].

Administration of TZDs (troglitazone, rosiglitazone, pioglitazone) is able to induce ovulation, to increase the ovulation rate and pregnancy in PCOS (cf [32]). For example, a large trial performed on 305 women has shown spontaneous ovulation in over 50% of the time (600 mg troglitazone) in comparison with approximately 10% of placebo group [33]. Troglitazone [33–36], pioglitazone [37–40], and rosiglitazone [41–43], for at least 3 months of treatment, improved insulin sensitivity, decreased the insulin concentration and reduced the androgenic activity. In these studies, a decrease in total and free circulating androgen concentrations associated with an increase of sex hormone binding globulin, SHBG, levels was observed. Concentrations of progesterone in serum are equivalent [35, 37], whereas those of estradiol are equivalent or decreased [36] after TZDs treatment. The body mass index is not significantly changed with the three TZDs [35–37, 39, 43, 44].

Furthermore, in PCOS patients with a “resistance” to antiestrogens (such as clomiphene citrate), an association of clomiphene citrate with TZDs (troglitazone, rosiglitazone) can help to increase the ovulation rate [45, 46]. Thus, TZDs, by an unknown mechanism (direct or indirect actions on hypothalamo-pituitary axis in order to remove the negative feedback of estradiol) could improve the clomiphene citrate sensitivity in PCOS patients.

TZDs treatment improves the rate of spontaneous pregnancy in several trials (20–40% pregnancy success) [33, 41, 44, 45, 47]. We can note that pioglitazone and rosiglitazone are both classified by the FDA (food and drug administration) as pregnancy category C and present potential teratogenic risks. PPAR*γ* is important for embryonic development [48] and TZDs can cause a decrease in the fetal maturation [49]. Nevertheless, two reported cases of human exposure to rosiglitazone during pregnancy have shown no malformation on babies [50, 51]. Despite this observation, women treated with TZDs will be stopped as soon as they will be pregnant. Similarly, preliminary studies have revealed that metformin, an insulin sensitizer more studied than TZDs, reduces also pregnancy losses, which are frequently observed (30–50%) in PCOS women during the first trimester [52, 53]. No risk for the fetus or teratogenicity was described after metformin administration (category B). However, it appears premature to maintain currently such treatments during pregnancy since there is no formal consensus about such indication [54].

## TZDS DID NOT SEEM TO AFFECT GONADOTROPIN SECRETION

In most trials, after TZDs treatment (troglitazone, rosiglitazone or pioglitazone), basal gonadotropin levels or the luteinizing hormone (LH)/follicle stimulating hormone (FSH) ratio did not change with the therapy [33, 35, 37, 42, 43]. In addition, recently, no alteration of the LH pulse frequency and amplitude, as well as gonadotropin responses to GnRH, was observed after pioglitazone treatment, either with or without insulin infusion [55]. Nevertheless, in some trials, a decrease in the plasma luteinizing hormone concentrations was observed after troglitazone, rosiglitazone or pioglitazone treatment [36, 38, 39, 44].

## DIRECT ACTION OF TZDS ON OVARY

Several studies in ruminants have shown a direct effect of glucose or fatty acids on folliculogenesis. The ovulation rate is increased without modification of gonadotropin secretion as observed in case of flushing [56]. In this perspective, we cannot reject the hypothesis for a direct action of TZDs on ovary.

### PPAR*γ* did not modify folliculogenesis or ovulation rate in rodents

Activation of PPAR*γ* by administration of 1 mg of ciglitazone/day injected intraperitoneally during four weeks in rats [57] did not alter folliculogenesis or the number of corpus luteum. In mice, deletion of PPAR*γ* specifically in ovaries did not change folliculogenesis or ovulation rate but decreased the number of embryos implanted, probably due to a drop in progesterone secretion by the corpus luteum [58]. Moreover, in human, linkage studies have rejected a genetic association between the PPAR*γ* locus (3p25) and the birth of dizygotic twin [59].

### PPAR*γ* modify steroids secretion by granulosa and thecal cells

In vitro, the steroids secretions (androgens, progesterone, estradiol) are inhibited or stimulated (about 20%) by TZDs according to species or the status of the cell differentiation (follicular phase, before or after the preovulatory surge). For example, TZDs stimulated progesterone secretion by a mixture of granulosa, theca, and stroma human cells obtained from premenopausal/perimenopausal patients at the time of oophorectomy [60], and TZDs inhibited testosterone secretion (≈15% reduction, [60]), progesterone and estradiol by human granulosa cells (after hCG stimulation for in vitro fertilization) or by luteal-granulosa cells obtained from PCOS patients [61, 62]. Furthermore, TZDs inhibited in vitro, LH/insulin-stimulated androgens secretion by porcine thecal cells [63].

In any case, the inhibiting effect of TZDs, in human ovarian cells, is more due to a reduction in the activity of steroidogenic enzymes 3-beta-hydroxysteroid-dehydrogenase (3*β*-HSD) and aromatase, rather than an activation of PPAR*γ* on the promoters of the genes encoding these enzymes [61, 64].

Improved insulin sensitivity in ovary induced by TZDs could also favor the restoration of steroidogenesis to a normal status. Indeed, the responsiveness to FSH in human granulosa cells obtained in PCOS patients was enhanced by insulin after improvement of the insulin sensitivity induced by the pioglitazone treatment [65]. In addition, in preliminary results, pioglitazone and rosiglitazone increased by two- to three-fold the level of insulin receptor and insulin receptor substrate-1 in human ovarian cells [66].

## IMPROVEMENT OF THE METABOLIC STATUS BY TZDS INCREASES FERTILITY IN PCOS PATIENTS

Thus, TZDs can act on the ovary in order to regulate steroidogenesis by a direct action on theca and granulosa cells via PPAR*γ*. Nevertheless, the actions of TZDs on steroidogenesis are not drastic and are varied in function by the status of the cell differentiation (and species). It will be more probable that a general improvement (redistribution of the fat tissue, increased in insulin sensitivity and inhibition of hepatic gluconeogenesis) stimulates ovulation through multiple ovary-independant mechanisms. The observations described below are in favour for this indirect action of TZDs in the treatment of PCOS.

### TZDs increase insulin sensitivity

TZDs reduce insulin resistance by improving sensitivity to insulin, mainly in adipose tissue and muscle of PCOS patients [22, 67]. TZDs could stimulate glucose transporter expression and other proteins in the insulin pathway (cf [21]). Moreover, a decrease in the insulin resistance by TZDs could be explained by a redistribution of the triglycerides circulating or content in liver and skeletal muscle into the adipose tissue. These modifications are associated with a decrease in plasma free fatty acid and triglyceride concentrations [22, 68]. Free fatty acid and/or triglyceride concentrations are high in PCOS patients [69] and decrease after TZDs treatment (cf [70]).

With this improvement of the general status, spontanenous ovulation could be favored. For example, only a weight reduction by diet and exercise improved insulin sensitivity and led to restoration of normal cycles. A 10–15% weight reduction could reduce hyperandrogenism and restored ovulation in more than 75% of PCOS obese patients [71, 72].

### TZDs could decrease androgen synthesis by a fat tissue redistribution

TZDs can decrease the high free androgen activity by two mechanisms: an increase in SHBG levels in serum, leading to a decrease in free circulating androgen levels [39], an adipose tissue redistribution. In contrast to metformin, a long-term TZDs treatment increases the body fat mass due to an increase in the subcutaneous adipose tissue [73] and a decrease in the amount of visceral abdominal adipose tissue associated with a decrease in free fatty acid [74]. The visceral fat mass has been associated with high serum androgen concentrations and was closely related to insulin resistance in women with PCOS [75, 76]. Thus, the reduction in the amount of profound visceral abdominal adipose tissue could contribute to explain the decrease in the testosterone and estradiol production, and consequently the improvement of the gonadotrophins pulsatility.

### TZDs could restore adipokines secretion implied in reproduction

Not only adipose tissue is involved for an energy storage, but also adipocytes secrete also hormones (TNF*α*, leptin, adiponectin, resistin, etc) which help to maintain homeostasis. These hormones are also implied directly in the regulation of the fertility at each level of the hypothalamo-pituitary-gonads axis (cf [77]). Moreover, the increased mass of the adipose tissue in PCOS patients alters the hormonal secretion (higher circulating levels of TNF*α*, resistin and lower levels of adiponectin [78, 79]). However, TZDs-induced return to a “normal metabolic state” may lead to a normal hormonal secretion by adipocytes. TZDs via PPAR*γ* stimulate adipocyte differentiation and increase the number of smaller adipocytes that are highly insulin sensitive [80–82]. These small adipocytes produce fewer free fatty acids, TNF*α* and leptin [81]. In addition, TZDs stimulate adiponectin secretion by adipocytes in vitro [83], and adiponectin levels were increased in PCOS women treated by rosiglitazone [84]. Adiponectin sensitizes cells to insulin and inhibits resistin secretion by adipocytes, which antagonizes the insulin action [73, 85]. Furthermore, in vitro, human thecal cells stimulated previously by insulin or forskolin, and then treated with resistin, have shown an increased activity of the p450c17 enzyme, leading to a stimulation of the androgens secretion [86].

## COMPARISON BETWEEN METFORMIN AND TZDS

Metformin seems to improve fertility of PCOS patients and is commonly used as an adjuvant to general lifestyle improvements [87, 88]. Metformin acts by a decrease of peripheral insulin resistance but new results suggest that metformin can regulate directly folliculogenesis at the ovarian level. In vitro in rat hepatocytes or in vivo in the human skeletal muscle, metformin activates AMP-activated protein kinase (AMPK), a regulator of energy balance [89]. In rat granulosa cells, activation of AMPK-induced by metformin decreases progesterone secretion, and the levels of proteins implied in steroidogenesis (3*β*-HSD, CYP11a1, STAR, and CYP19A1) [90]. Moreover, in human granulosa cells, metformin decreases progesterone secretion [91] and androgens synthesis through a direct inhibition of the Cyp17 activity [92]. Recently, AMPK was described to be activated indirectly by TZDs and independently of PPAR*γ* [30]. These two insulin-sensitizing agents (metformin and TZDs) cause a rapid increase in the cellular ADP : ATP ratio, probably due by the inhibition of the respiratory chain, which can lead to the phosphorylation of AMPK [93].

Interestingly, PCOS women treated with metformin present lower follicular fluid concentrations of testosterone and insulin and after gonadotropin-stimulation for in vitro fertilization, the number of mature oocytes retrieved and oocytes fertilized was increased in comparison with controls [94].

Comparative studies [47, 95–97] performed between the two treatments (metformin and TZDs) have shown similar [47, 96] or better performance [96, 97] for TZDs to improve regular menstrual cyclicity (87.8% with rosiglitazone versus 79.3% with metformin, [96]), the ovulation rate and the pregnancy rate in PCOS patients.

The different mechanism used by metformin and by TZDs to improve fertiliy induces new possibility in the treatment of PCOS. For example, one clinical trial has tested co-administration of pioglitazone and metformin to PCOS women nonoptimally responsive to metformin. The percentage of menses increased two-fold after co-administration in comparison, with only metformin-treated women [98].

## CONCLUSION

Overall, insulin-sensitizing treatments for PCOS patients, such as metformin or TZDs, lead to a strong improvement of the fertility. These treatments have several sites of action (steroid secretion by ovarian cells; insulin sensitivity in muscle and adipocyte and fat redistribution). More and more clinical data are now available and encourage us to redefine our approach of insulin resistance, and the treatment of infertility in patients with PCOS.

Creation of promising ligand, for example a dual PPAR*α*/PPAR*γ* agonist (glitazar class) [99, 100], could be useful to treat insulin sensitivity, atherosclerotic vascular, and fertility in PCOS women. Nevertheless, before using it in routine clinical practice, several extended safety tests should be necessary to estimate the potential risk of these synthetic ligands.

It is also necessary to keep in mind that TZDs can present a risk to the fetus during pregnancy and therefore their use should be carefully monitored, especially during the first weeks of pregnancy. Finally the development of animal models, mimicking PCOS is probably mandatory in next few years to increase our knowledge on this syndrome and to better understand the molecular actions of metformin and TZDs in target organs.
